# The diagnosis and treatment for a patient with cancer of unknown primary: A case report

**DOI:** 10.3389/fgene.2023.1085549

**Published:** 2023-01-19

**Authors:** Hong Hu, Qin Pan, Jiaying Shen, Junlin Yao, Guoxiang Fu, Fengjuan Tian, Na Yan, Weidong Han

**Affiliations:** ^1^ Department of Medical Oncology, Qiantang Campus of Sir Run Run Shaw Hospital, College of Medicine, Zhejiang University, Hangzhou, Zhejiang, China; ^2^ Department of Medical Oncology, Sir Run Run Shaw Hospital, College of Medicine, Zhejiang University, Hangzhou, Zhejiang, China; ^3^ Department of Pathology, Sir Run Run Shaw Hospital, College of Medicine, Zhejiang University, Hangzhou, Zhejiang, China; ^4^ Department of Radiology, Sir Run Run Shaw Hospital, College of Medicine, Zhejiang University, Hangzhou, Zhejiang, China; ^5^ Key Laboratory of Digital Technology in Medical Diagnostics of Zhejiang Province, Dian Diagnostics Group Co., Ltd., Hangzhou, Zhejiang, China

**Keywords:** cancer of unknown primary, traceability of tumor tissue, gene expression profile, lung metastasis of breast cancer, case report

## Abstract

**Background:** Cancer of unknown primary (CUP) is a class of metastatic malignant tumors whose primary location cannot be determined. The diagnosis and treatment of CUP are a considerable challenge for clinicians. Herein, we report a CUP case whose corresponding primary tumor sites were successfully identified, and the patient received proper treatment.

**Case report:** In February 2022, a 74-year-old woman was admitted to the Medical Oncology Department at Sir Run Run Shaw Hospital for new lung and intestinal tumors after more than 9 years of breast cancer surgery. After laparoscopically assisted right hemicolectomy, pathology revealed mucinous adenocarcinoma; the pathological stage was pT2N0M0. Results from needle biopsies of lung masses suggested poorly differentiated cancer, ER (-), PR (-), and HER2 (-), which combined with the clinical history, did not rule out metastatic breast cancer. A surgical pathology sample was needed to determine the origin of the tumor tissue, but the patient’s chest structure showed no indications for surgery. Analysis of the tumor’s traceable gene expression profile prompted breast cancer, and analysis of next-generation amplification sequencing (NGS) did not obtain a potential drug target. We developed a treatment plan based on comprehensive immunohistochemistry, a gene expression profile, and NGS analysis. The treatment plan was formulated using paclitaxel albumin and capecitabine in combination with radiotherapy. The efficacy evaluation was the partial response (PR) after four cycles of chemotherapy and two cycles combined with radiotherapy.

**Conclusion:** This case highlighted the importance of identifying accurate primary tumor location for patients to benefit from treatment, which will provide a reference for the treatment decisions of CUP tumors in the future.

## Introduction

Cancer of unknown primary (CUP) is a malignant tumor whose primary focus cannot be determined after detailed examination and evaluation. It accounts for 1–5% of all malignancies (Fizazi et al., 2015). A small number of CUP patients (15–20%) can be inferred from primary tissue according to clinical and histopathological results, and the prognosis is usually better with corresponding tumor-type-specific treatment (Li et al., 2019; Rassy and Pavlidis, 2020). However, the majority of CUP patients cannot identify the primary tumor. The median survival time of these patients is only 4.5 months, the 1-year survival rate is 20%, and the 5-year survival rate is only 4.7% (Richardson et al., 2015; Pu et al., 2021). The current standard treatment for CUPs is based on the primary site, so they can only receive empirical chemotherapy and have a poor prognosis (Pavlidis, 2007; Daud, 2013; Kim et al., 2018a). In recent years, molecular detection in tumor tissue tracing has shown that gene expression profiling can be used to trace the location of primary tumors by detecting the expression level of specific genes in tumor tissue (Dermawan and Rubin, 2021; Liu et al., 2021; Chen et al., 2022). Here, we report a CUP patient who sank into an unfavorable subset with lung and intestinal tumors. The location of primary tumor tissue was inferred based on molecular expression profile analysis. Then, the treatment plan was formulated using immunohistochemical (IHC) analysis and NGS analysis. The patient obtained clinical benefits from the combination of chemotherapy and radiotherapy.

## Case presentation

**FIGURE 1:**
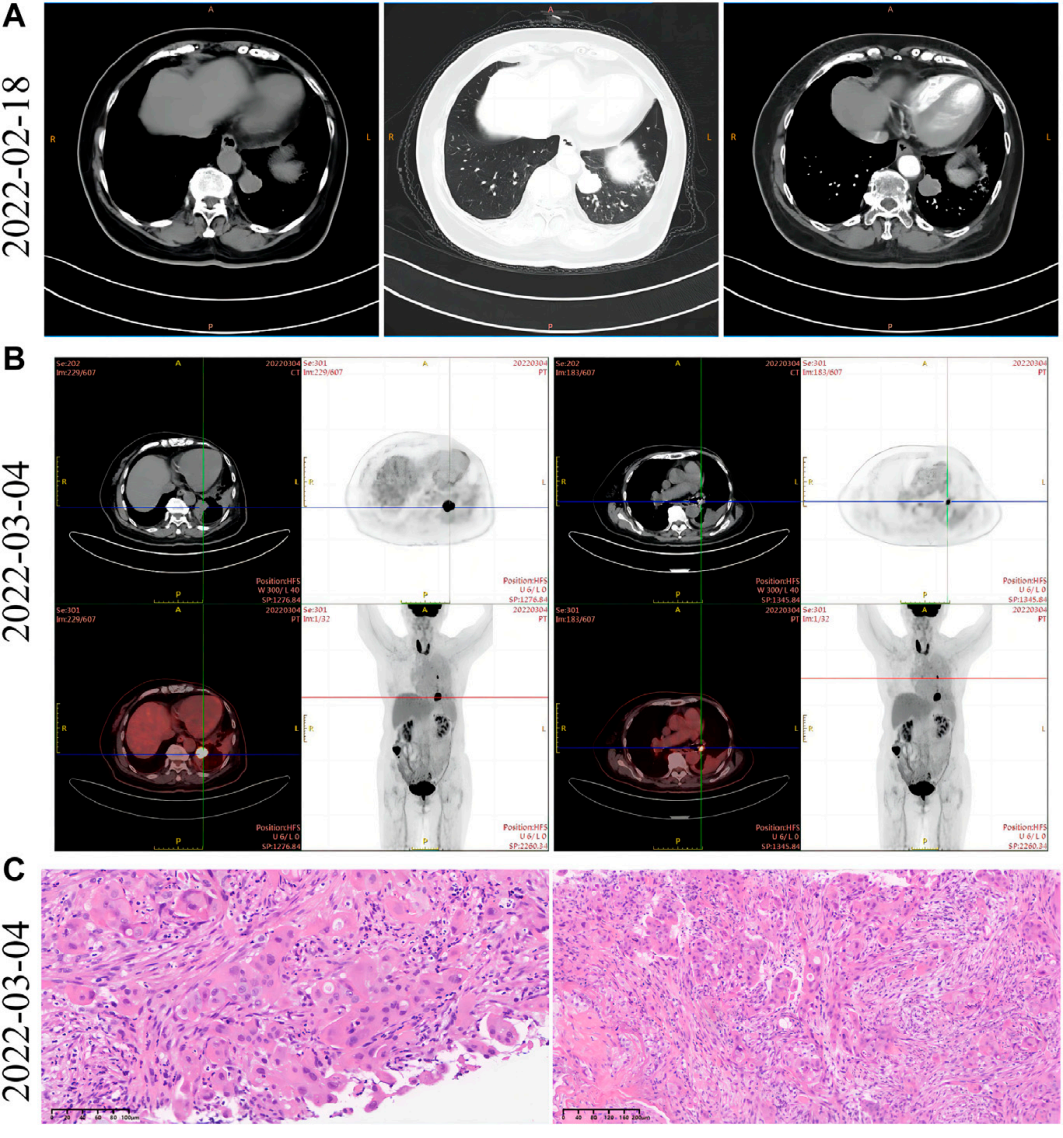
Imaging and pathological data of lung lesions at initial diagnosis. **(A)** Left side: the contrast-enhanced mediastinal chest CT window showed a mass of high-density shadow in the left lower lobe of the lung. Middle: the pulmonary window in the arterial phase of chest contrast-enhanced CT revealed a shallow 25-mm lobular mass in the left lower lobe. The boundary was clear and regular. Right side: the mediastinal arterial window of chest contrast-enhanced CT revealed a mass of high-density shadow in the left lower lobe that exhibited mild-to-moderate progressive enhancement and noticeable marginal enhancement. **(B)** Left side: PET-CT showed a soft tissue mass in the left lower lobe of the lung. It increased FDG uptake, and the maximum SUV is 15.57. Right side: an enlarged lymph node with increased FDG metabolism is observed below the left pulmonary hilum. The diameter is about 1 cm, and the maximum SUV is 4.38. **(C)** Pathological analysis of tumor biopsies in the left lower lobe of the lung. The morphology of tumor cells was observed after staining with H&E.

**FIGURE 2:**
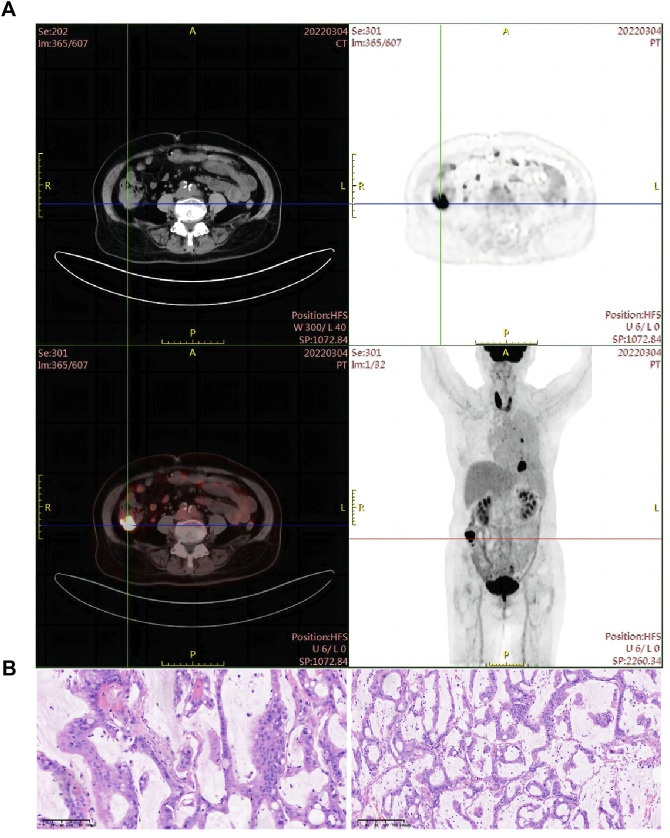
Imaging and pathological data of ascending colon lesions. **(A)** PET-CT of ascending colon lesions. There was more content in the ascending colon, and the local nodular FDG metabolism was increased. SUV_max_ = 12.61 for early imaging, and SUV_max_ = 14.11 for delayed imaging. **(B)** Pathological analysis of tumor biopsy in ascending colon lesions. The morphology of tumor cells was observed after staining with H&E.

**FIGURE 3:**
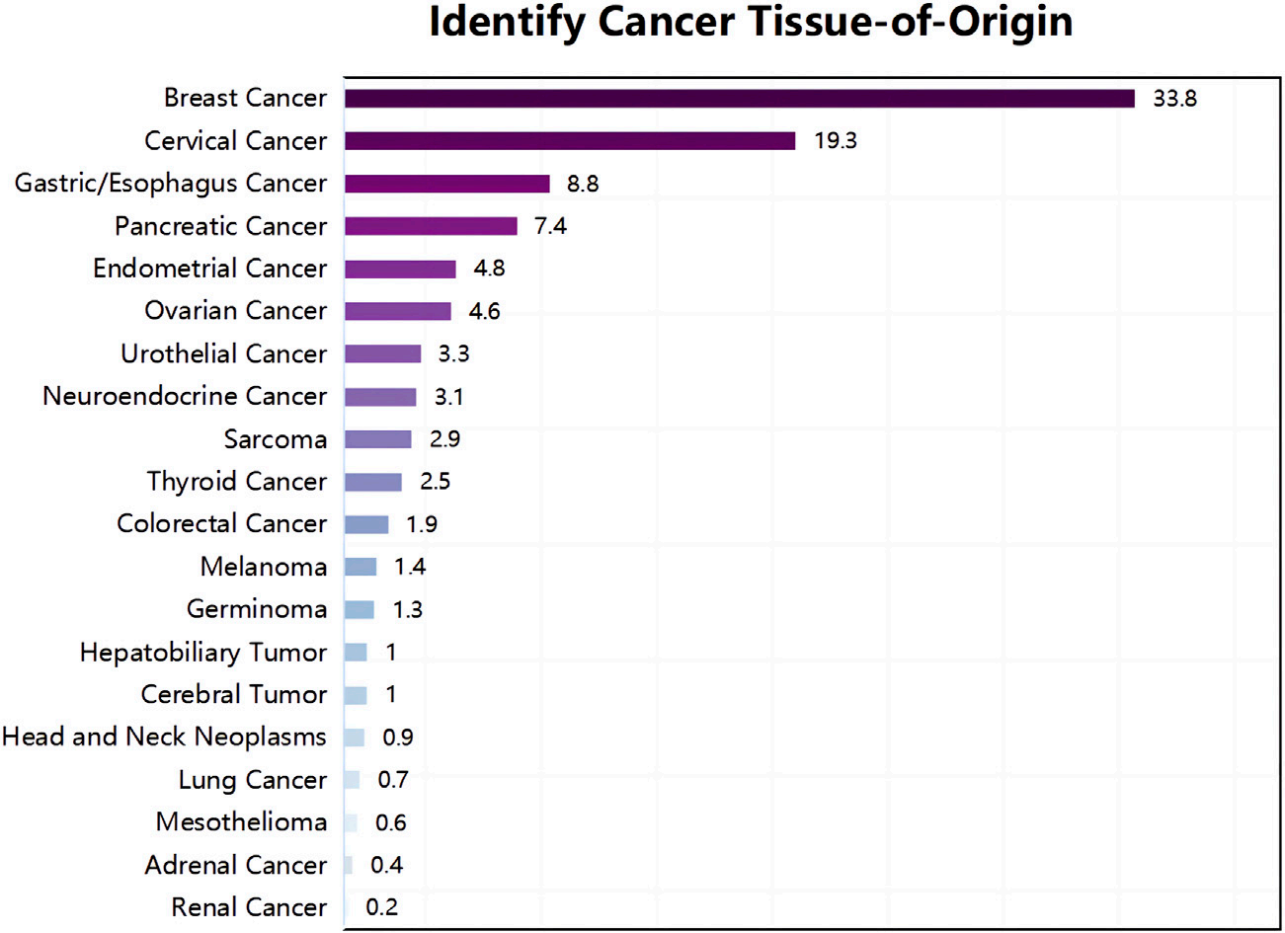
Tumor tissue traceability expression assay results. The maximum similarity score for tumor tissue traceability was 100.

**FIGURE 4:**
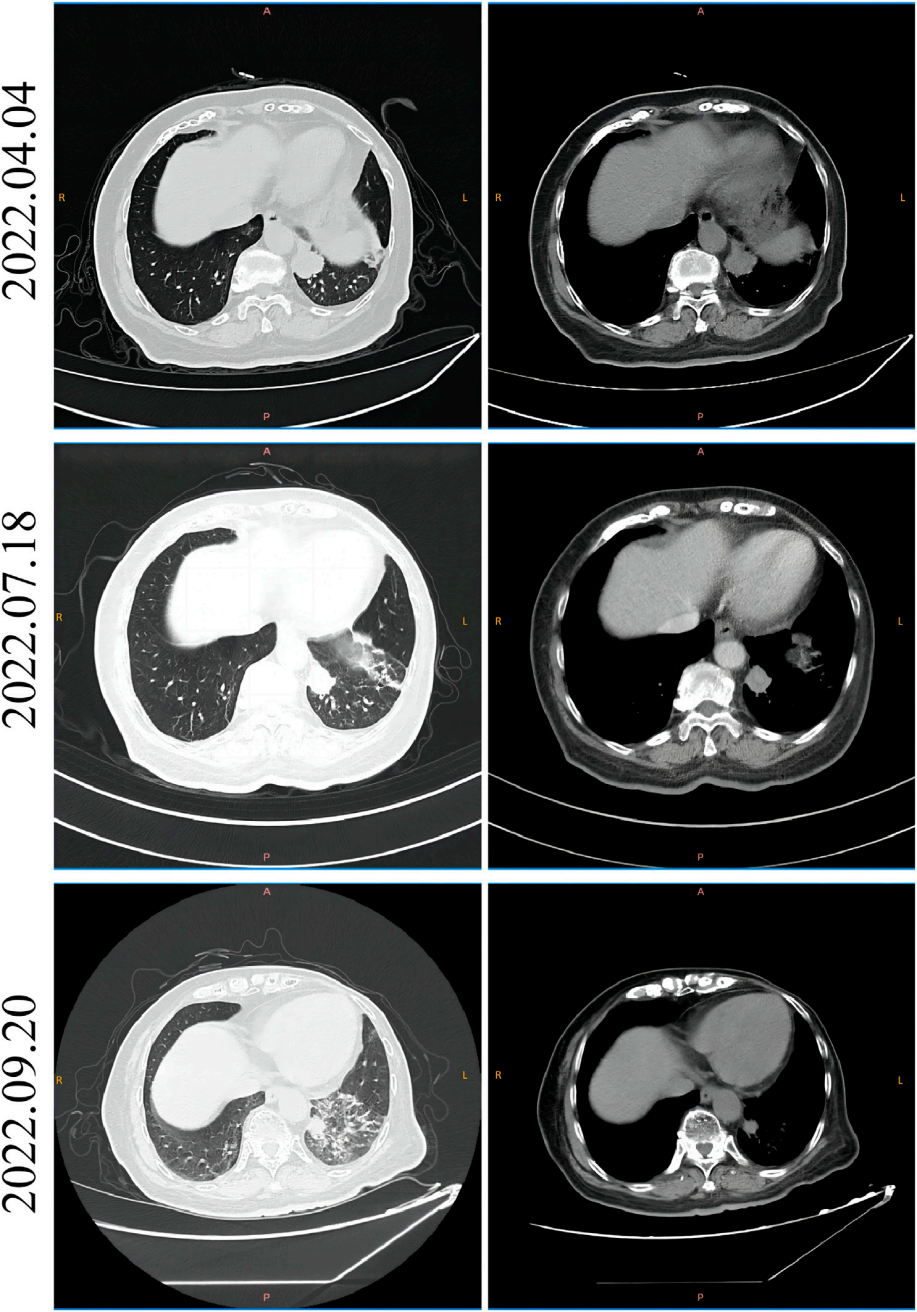
CT imaging of the patient before and after the initial chemotherapy and radiotherapy. The left side is the lung CT scan window, and the right side is the mediastinum CT scan window. The CT image of the patient before receiving chemotherapy on 4 April 2022; the CT image of the patient receiving chemotherapy but not radiotherapy on 18 July 2022; and the CT image of the patient receiving chemotherapy and radiotherapy on 20 September 2022.

In February 2022, a 74-year-old woman was presented with a mass shadow in the lower lobe of her left lung during a periodic review after a breast cancer operation. (Figure 1A). This patient underwent a left mastectomy in September 2012 due to a malignant tumor in the left breast in another hospital. Postoperative pathology showed that the malignant tumor in the left breast was an invasive lobular carcinoma. Metastatic cancer was not found in lymph nodes and did not metastasize in pectoralis fibro-fatty tissue. Tumor cells appeared as cords and trabeculae with fibrous tissue hyperplasia (Supplementary Figure S1). A series of IHC results suggested ER (+), PR (-), HER2 (-), Ki-67 (+, 5%), E-cadherin (-), P120 (+), EGFR (-), CK5/6 (-), P63 (-), calponin (-), CK7 (+), CK20 (-), LCA (-), TTF-1 (-), HEP (-), GCDFP-15 (-), PAS (+), and AB (-). The patient was treated with six cycles of anthracycline drugs after surgery, and exemestane was prescribed until December 2021, during which the periodic review showed no abnormalities. The CT scan found a pulmonary nodule in the left lung until February 2022. The abnormal focus of the lung was on the left side of the body, which was the same as the breast lesions 9 years ago. PET-CT showed that the nodules in the left lower lobe of the lung were well demarcated and had morphological rules that were not typical of lung cancer (Figure 1B). On 4 March 2022, the left lower lobe tumor biopsy of the patient’s lung showed poorly differentiated carcinoma (Figure 1C). The combined results of differential IHC detection for lung adenocarcinoma and squamous cell carcinoma suggested TTF-1 (-), napsin A (-), CK7 (+), p40 (-), and p63 (-). The results of IHC detection in the left lower lobe of the lung to analyze whether it originated from breast cancer suggested ER (-), PR (-), HER2 (-), Ki-67 (+, 15%), GCDFP-15 (-), and GATA-3 (-). The results of IHC detection in the left lower lobe of the lung to analyze whether it originated from intestinal cancer suggested CK20 (-), SATB2 (-), and CDX-2 (-). PD-L1 (22C3) showed a TPS<1%. Tumor markers were all in the normal range, including CA125, CEA, SCC, NSE, ProGRP, and CYFRA21-1. Practitioners considered not to exclude metastatic breast cancer through a combination of clinical history, IHC analysis, and imaging manifestations. Furthermore, PET-CT was implemented to understand the patient’s general condition. Results showed that a malignant tumor is considered in the lower lobe of the left lung, and lymph node metastasis should be considered under the left pulmonary hilum (Figure 1B). There were no space-occupied lesions and abnormal metabolism of FDG in the postoperative area of left breast cancer. It was worth noting that PET-CT found a malignant space-occupying lesion in the initial segment of the ascending colon (Figure 2A). The subsequent colonoscopic biopsy revealed mucinous adenocarcinoma. IHC results suggested GATA-3 (-), mammaglobin (-), CDX-2 (+), SATB2 (+, individual cells are positive), GCDFP-15 (-), and HER2 (-). Pathological analysis after laparoscopically assisted right hemicolectomy confirmed mucinous adenocarcinoma of the colon again (Figure 2B). The pathological stage was pT2N0M0, which was different from the pathology of the left inferior pulmonary lobe. At this point, the origin of the malignant lung mass in this patient had not been determined after a pathological assessment of needle biopsy and imaging evaluation. A surgical pathology sample was needed to determine the origin of the tumor tissue, but the patient’s chest structure showed no indications for surgery. We used two simultaneous approaches to identify the source of malignant tumors in the lower lobe of the left lung. The tissue traceability expression profile was initially detected in punctured tissue, and the details are provided in Supplementary Table S1. The results showed that the malignant tumor in the lower lobe of the left lung originated from breast cancer (Figure 3). The maximum tumor tissue similarity score was 100, and the results suggested that lung and colorectal cancer similarity scores were deficient, at only 0.7 and 1.9, respectively. It indicated that the lesion in the lower lobe of the left lung was not a primary pulmonary lesion and was not metastatic from the ascending colon. This was consistent with the pathological analysis results. The left lower lobe nodule was a poorly differentiated carcinoma, and the ascending colon nodule was a mucinous adenocarcinoma. There were no apparent standard features between the two lesions. The tissue traceability expression profile results exhibited essential reference values for subsequent diagnosis and treatment. Then, more than 600 hot spot exon regions and some intron regions of tumorigenesis and developmental genes were analyzed by NGS. DNA was extracted from plasma. The Illumina NextSeq 500 System (Illumina, San Diego, USA) was used for NGS. The average sequencing depth was at least ×1,000. Unfortunately, no tumor-driving gene was found in this test. Only a somatic point mutation FAT1 p.G4570S was obtained, which has not been found in other studies and has not been recorded in COSMIC or other databases. Based on the comprehensive evaluation results, a malignant lung tumor in the left lower lobe was initially diagnosed as stage IV triple-negative cancer. After a multidisciplinary treatment (MDT) discussion, the patient was treated with C1–C4 paclitaxel albumin and capecitabine on 2022-4-7, 2022-4-29, 2022-5-24, and 2022-6-20. The specific regimen was planned with 1,500 mg orally twice daily on days 1–14 and 200 mg intravenous infusion of paclitaxel albumin on days 1 and 8. The radiotherapy plan was added on 22 July 2022, including 10 MV-X SAD 100 DT 214 cGy/F/d in the intensity-modulated field of the chest tumor area (including lesions in the lower left lung and mediastinal metastatic lymph nodes) and 10 MV-X SAD 100 DT 180 cGy/F/d in the intensity-modulated field of the high-risk chest area. She was scheduled to finish radiotherapy 28 times. Combined with the chemotherapy regimen, the specific regimen was provided with 1,500 mg orally twice daily and 190 mg intravenous infusion of paclitaxel albumin on days 1 and 8 (q3w). The longest diameter of the left lower pulmonary nodule narrowed from 29 mm to 14 mm in September 2022. A partial response (PR) was achieved, with lesion diameters decreasing by 51.72% (Figure 4). She is currently in stable condition.

## Discussion

CUP was divided into two subgroups according to their clinical and pathological conditions in traditional assessment (Pavlidis et al., 2003). For the first subgroup, 15–20% of patients showed specific clinical and pathological characteristics, which strongly indicate the origin of tumor tissue (El Rassy et al., 2019; Rassy et al., 2019; Rassy et al., 2020a). This part of CUP has chemosensitive tumors with good prognoses when treated with native treatments (Fizazi et al., 2015). However, 80–85% of patients fall into the second subtype. This subtype cannot identify the primary tumor location and is managed using empirical chemotherapy, which has a poor prognosis (Alvi et al., 2017). It should be noted that there are obvious deficiencies in previous studies comparing the prognosis assessment of site-specific therapy with empirical chemotherapy. These deficiencies include patient accrual problems, study design limitations, heterogeneity among CUP classifiers, and incomparable treatments. Elie et al. recommended two comprehensive clinical trial designs, namely, visionary and pragmatic approaches that can implement the latest diagnostic and therapeutic advances to improve the prognosis of CUP patients.

It is gratifying to note that the subgroup with a poor prognosis can increase survival rates through histological diagnostic tools, site-specific treatment, and new therapeutic exploration (Mikhail et al., 2015). The primary tumor focus is helpful for physicians to design more targeted treatment plans, which are essential to identifying them accurately (Jardim et al., 2015; Schwaederle et al., 2015). With the increasing availability of high-throughput genomic and transcriptomic data, molecular biomarkers in The Cancer Genome Atlas are gradually enriched, including somatic mutation (Fernandez et al., 2012; Rassy et al., 2020b; Chebly et al., 2022), copy number variation (CNV) (Gatalica et al., 2018; Naing et al., 2020), gene expression (Rassy et al., 2021), microRNA expression, and DNA methylation (Bettegowda et al., 2014), which can be used to identify the primary cancer focus. Some studies have shown that the gene expression profile of the metastatic tumor is different from the tissue at the metastatic site and more similar to its primary site (Kim et al., 2018b; Gandara et al., 2018; Kim et al., 2022). It has been suggested that cancer always retains the gene expression characteristics of its tissue origin in tumor occurrence, development, and metastasis. Other studies have found that cancer cells are characterized by a massive overall loss of DNA methylation and that different tumor tissues have different epigenetic signatures depending on the tissue (Fernandez et al., 2012). DNA methylation is an effective biomarker for clinical diagnosis and can distinguish other tissues of origin (31). In addition, Küsters-Vandevelde et al. have found that specific CNVs may be associated with cancer metastasis (Naing et al., 2020). Liu et al. (2021) systematically compared the performance of three biomarkers (DNA methylation, gene expression profile, and somatic mutation) and their combinations in estimating the tissue origin of patients with CUP. The results showed that the accuracy of the gene expression profile was the highest, followed by DNA methylation, and the accuracy of somatic mutation was the worst of the three. However, Elie Rassy et al. (Rassy and Pavlidis, 2020) found that the prediction accuracy of methylation was 78%–87% and the gene expression profile was 54%–98%. More research is needed to explore which method is more accurate in identifying the origin of CUP. Positive hints have also appeared in the exploration of novel CUP therapies. A large study (32) demonstrated that the molecular patterns of BRAF/NRAS alterations were similar between patients with melanoma of unknown primary site and stage-matched melanoma of known prior site. Therapy with RAF/MEK inhibitors may improve survival for MUP. Chromosomal instability (CIN) has been considered a characteristic of some cancer cells but is not frequent in CUP (Rassy et al., 2020b; Chebly et al., 2022). It may provide therapeutic benefit from immune checkpoint inhibitors (ICIs) in patients with CUP. In addition, 28% of patients with CUP displayed one or more predictive biomarkers to ICI, such as PD-L1 expression on ≥5% cancer cells at 22.5% (≥1% at 34%) and lymphocytes at 58.7%, high microsatellite instability (MSI) at 1.8%, and tumor mutational burden (TMB) ≥17 mutations per megabase at 11.8% (Gatalica et al., 2018). A phase II trial, NCT02721732, showed that the clinical benefit rate of pembrolizumab for CUP patients was 54% (Naing et al., 2020).

In this case, the patient was confirmed to have a malignant nodule in the lower lobe of the left lung after a detailed case history, imaging, and cytopathological evaluation. However, its tissue origin could not be determined. Practitioners used lung puncture tissue samples to trace the source of tumor tissue by detecting 90 genes that are explicitly expressed in neoplastic tissue. Results from the gene expression profile showed that the malignant nodule in the left lower lobe of the lung originated from breast cancer but not from the lung where it occurred. At the same time, it also denied that it was a malignant tumor metastasis of the ascending colon. A review of postoperative pathological IHC outcomes in patients was performed 9 years ago, which suggested ER (+), PR (-), and HER2 (-). However, the histopathological indices of the left inferior pulmonary lobe were ER (-), PR (-), and HER2 (-). Through multidisciplinary discussion and literature research, we learned that ER, PR, and HER2 levels in primary lesions differed in metastatic lymph nodes and recurrent metastatic lesions. Changes in ER and PR levels may be related to the age of the initial onset and secondary endocrine therapy. In this case, exemestane was administered after primary lesion surgery until December 2021. It may be one of the underlying causes of triple-negative malignant nodules in the left lower lobe of the lung.

To find a personalized treatment schedule for this case, we conducted a high-depth sequencing analysis of circulating blood-derived tumor DNA (ctDNA). Unfortunately, next-generation sequencing has not detected effective drug targets. A previous study showed that CUP patients with TMB >10 mutations/Mb had a better prognosis trend when receiving ICI treatment. The TMB assessment, in this case, was 0.7 mutations/Mb (Rassy et al., 2021). It should be noted that this patient was bleeding during the second puncture, so further sampling was stopped. The evaluation of the patient’s chest structure revealed that it was not suitable for surgery. The blood sample was taken for testing. ctDNA in the blood was obtained from the release of tumor tissue. If tissue samples cannot be obtained for advanced-stage patients, ctDNA may be used for detection (Bettegowda et al., 2014; Kim et al., 2022). TMB can be accurately and repeatedly measured in plasma, which has been confirmed on the basis of POPLAR (NCT01903993) and OAK (NCT02008227) studies (Gandara et al., 2018). bTMB was positively correlated with the effectiveness of immune checkpoint inhibitors. B-F1RST research proved that patients with bTMB-H (bTMB ≥16) obtained clinical benefits in PFS, ORR, and OS (Kim et al., 2018b; Socinski et al., 2019). However, bTMB is still in the exploration stage. Currently, the tissue specimen is the preferred type for TMB detection. We developed a triple-negative breast cancer treatment plan for this case: paclitaxel albumin combined with capecitabine. It was gratifying that the malignant tumor in the left lower lobe of the lung was in remission under this treatment regimen. Next-generation ctDNA sequencing identified an unreported FAT1 p.G4570S mutation whose clinical significance was unknown. Some studies have suggested that mutations in the *FAT1* gene accelerate tumorigenesis and malignant progression and promote the transformation of epithelial cells into mesenchymal cells (38, 39). FAT1 p.G4570S occurred in intrinsically disordered regions (IDRs) that require further research to explore the role in biological functions.

In summary, we present a patient with CUP whose location of tumor origin was successfully identified based on the results of a multiplex gene expression profiling analysis, and the patient benefited from specific treatment according to the tissue source. This case suggests that multiple gene expression profiling may be considered to assist in diagnosing tissue origin in CUP patients when conventional clinical testing methods fail to determine the source of tumor tissue. We believe that accurate diagnosis and specific CUP treatment are essential to improving patient outcomes.

## Data Availability

The original contributions presented in the study are included in the article/supplementary material. Further inquiries can be directed to the corresponding authors.
