# Supplementary material for: The diagnosis and treatment for a patient with cancer of unknown primary: A case report
Source: Front Genet. 2023 Jan 19;14:1085549. doi: 10.3389/fgene.2023.1085549 (PMC9894331; doi:10.3389/fgene.2023.1085549)
Supplement: Supplementary file 1 [file Table1.DOCX]

[The Diagnosis and Treatment for a Patient with Cancer of Unknown Primary: A Case Report.](https://pubmed.ncbi.nlm.nih.gov/34887715)

**SUPPLEMENTARY DATA**

1. **90-Gene Classifier for Tumor Classification: Brief Introduction and Gene List.**

Cancer-specific gene markers were identified by a pan-cancer transcriptome database of 5434 specimens representing 21 tumor types. The database included primary and metastatic tumors and well-differentiated to undifferentiated tumors. The SVM-RFE (Support Vector Machine-Recursive Feature Elimination) machine learning algorithm was used to select the top 10 predictive genes for the 21 tumor types. After removing redundant genes, a list of 90 genes specific to 21 tumor types was identified. Then, an SVM linear model was trained by the entire pan-cancer transcriptome database to form a multi-class classification algorithm. Mathematically, the 90-gene classifier created a hyperplane for each tumor type in 90-dimensional space. The algorithm calculates its 90 gene expression values for an unknown test sample, projects it into 90-dimensional space, and estimates its distance to the 21 hyperplanes. The position of the test sample relative to the hyperplane determines its membership in one class or another (e.g., "breast cancer" vs. "not breast cancer"). In addition, the confidence of the test sample belonging to a tumor type is proportional to the distance of the test sample from the corresponding hyperplane. Then, the lengths of the test sample from each of the 21 hyperplanes were compared and converted to similar scores with the Platt Scaling formula. Intuitively, similarity scores reflect the similarity degree of the test sample's gene expression pattern. Similarity scores were probability-based, with a reported range of 0 to 100. The tumor type with the highest similarity score was defined as the predicted tumor type by the 90-gene classifier.

- 1. Gene List

| ACPP | COL11A1 | IGFBP7 | NPTX2 | SERPINA3 |
| --- | --- | --- | --- | --- |
| ACTG2 | CXCL14 | IGJ | NPY1R | SERPINB3 |
| AGR2 | CYP17A1 | ISL1 | PCDH7 | SFN |
| APOBEC3B | DLK1 | KLK2 | PCP4 | SFRP1 |
| APOD | EPCAM | KLK3 | PEG3 | SFTPB |
| ASPN | ESR1 | KRT13 | PI15 | SLC3A1 |
| ATP1B1 | FABP1 | KRT14 | PIGR | SPINK1 |
| AZGP1 | FABP4 | KRT15 | PLA2G2A | SPP1 |
| C7 | GATA3 | KRT19 | POSTN | SST |
| CA12 | GCG | KRT20 | PRRX1 | SULT2A1 |
| CDH1 | GFAP | LGALS4 | PTGDS | TACSTD2 |
| CDH17 | GJA1 | LUM | PTN | TG |
| CEACAM5 | GPM6B | MGP | RPS11 | TH |
| CEACAM6 | GPX3 | MMP1 | RPS4Y1 | TM4SF4 |
| CHGA | GREM1 | MMP12 | S100A2 | TSPAN8 |
| CHI3L1 | HBB | MMP3 | S100A8 | TYRP1 |
| CLDN18 | ID4 | MSMB | S100P | VEGFA |
| CLU | IGFBP2 | NKX3-1 | SCGB2A2 | XIST |

- 1. Sample Preparation and RNA Isolation

Five to fifteen 5mm unstained sections were freshly cut for gene expression analysis. FFPE tissue samples were centralized, and H&E-stained slides from the case were reviewed to assess the percentage of tumor cells and necrotic areas by two senior FUSCC pathologists. Tumor tissue regions were marked on H&E stained slides and manually macrodissected for tumor cell enrichment. Total RNA isolation and gene expression profiling were performed at Canhelp Genomic Reference Laboratory (Hangzhou, China). Total RNA was extracted using a protocol-based Total RNA Isolation Kit (Canhelp, Hangzhou, China). Briefly, FFPE tissue was deparaffinized, followed by digestion, DNase treatment, and total RNA elution. Total RNA concentration was measured by spectrophotometer at 260 nm absorbance, and purity was quantified using the A260/A280 ratio. RNA sample with A260/A280 ratios between 1.7 and 2.1 was enrolled in the next step experiment.

- 1. Expression profiling of 90 tumor-specific genes

As previously described, qRT-PCR was used to measure gene expression levels of 90 tumor-specific genes. For each sample, reverse transcription was performed on isolated total RNA by a High Capacity cDNA Reverse Transcription Kit with RNase Inhibitor (Applied Biosystems, Foster City, CA, United States). Subsequently, qRT-PCR was performed using a 7500 Real-Time PCR system (Applied Biosystems) to measure the expression levels. PCR cycling conditions were 10 min at 95℃ and 40 cycles of 15 s at 95℃, and 1 min at 60℃.

- 1. Data analysis

Gene expression data were analyzed using R software (version 3.6.0) and Bioconductor product packages (version 3.9). The gene expression pattern for each sample was compared with the 21 tumor types indicated by the 90-gene expression signature. Similarity scores were calculated for the 21 tumor types, which showed similarities in the gene expression pattern between the sample and tumor type. Similarity score values ranged from 0 (very low similarity) to 100 (very high similarity), which summed up to 100 across all 21 tumor types. The tumor type with the highest similarity score was considered to indicate the tissue of origin.
