# Supplementary figures and images for: The diagnosis and treatment for a patient with cancer of unknown primary: A case report
Source: Front Genet. 2023 Jan 19;14:1085549. doi: 10.3389/fgene.2023.1085549 (PMC9894331; doi:10.3389/fgene.2023.1085549)

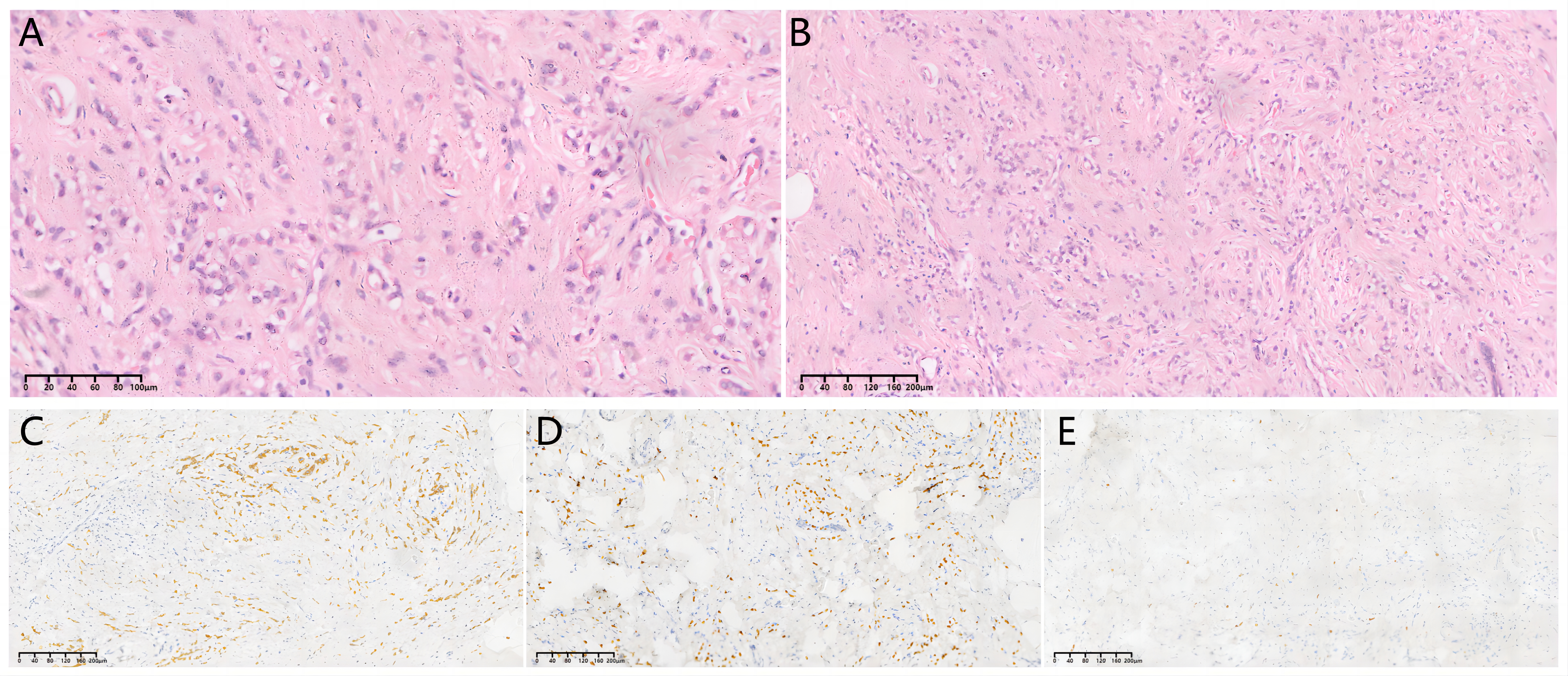

Supplement: Supplementary file 2 [file Image1.JPEG]
